# Supplementary material for: Nitric Oxide Participates in Aluminum-Stress-Induced Pollen Tube Growth Inhibition in Tea (Camellia sinensis) by Regulating CsALMTs
Source: Plants (Basel). 2022 Aug 29;11(17):2233. doi: 10.3390/plants11172233 (PMC9460577; doi:10.3390/plants11172233)
Supplement: Supplementary file 1 [file plants-11-02233-s001.zip › plants-1863405-supplementary.pdf]

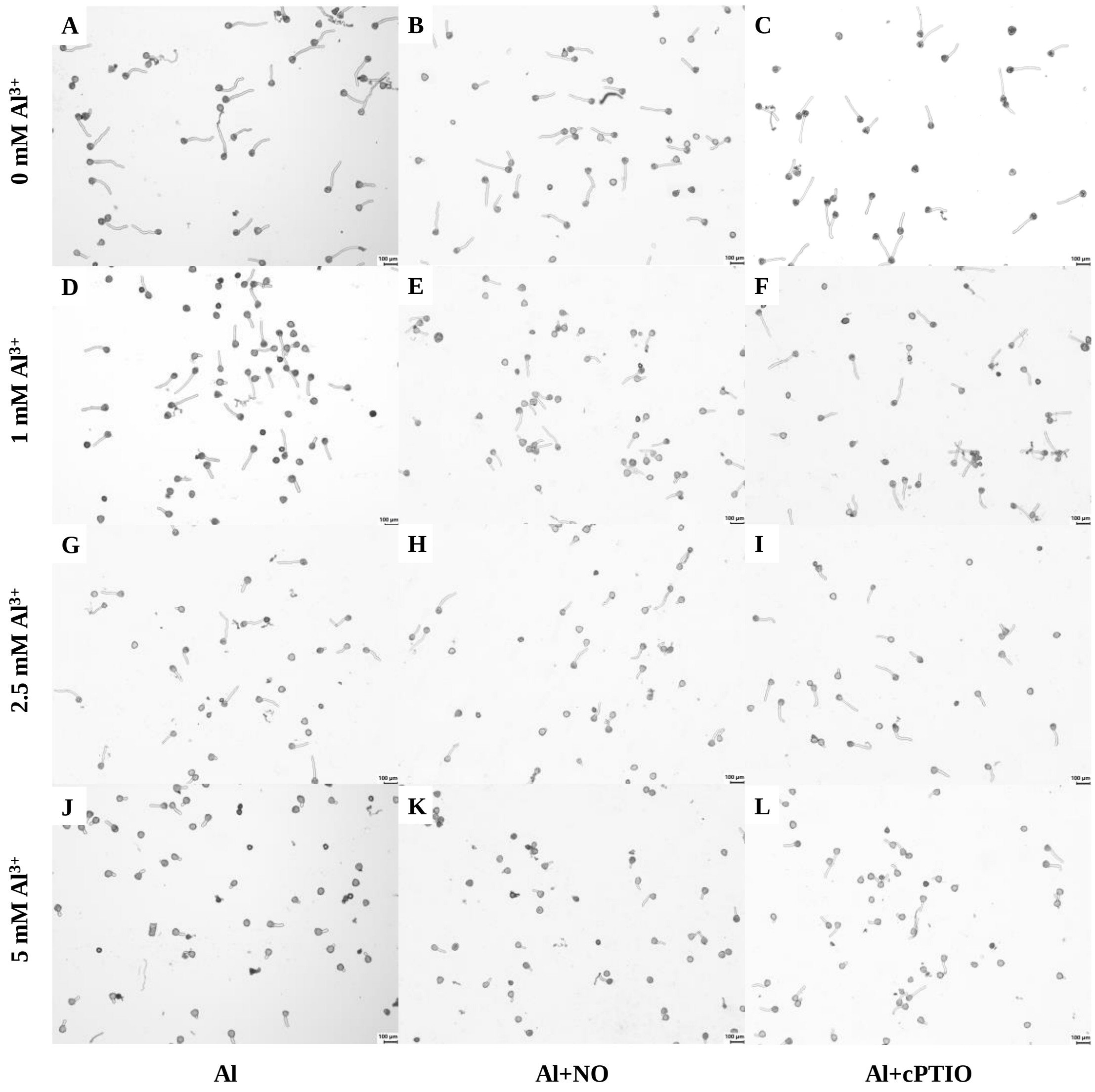

Figure S1 The phenotypes of *Camellia sinensis* pollen tubes under different treatments. AT represent the treatment group which treated with Al concentration gradient only, NT represent the treatment group which treated with Al^{3+} concentration gradient treatment and 25 μM DEA NONOate (NO donor), CT represent the treatment group which treated with Al^{3+} concentration gradient treatment and 200 μM carboxy PTIO potassium salt (NO scavenger).

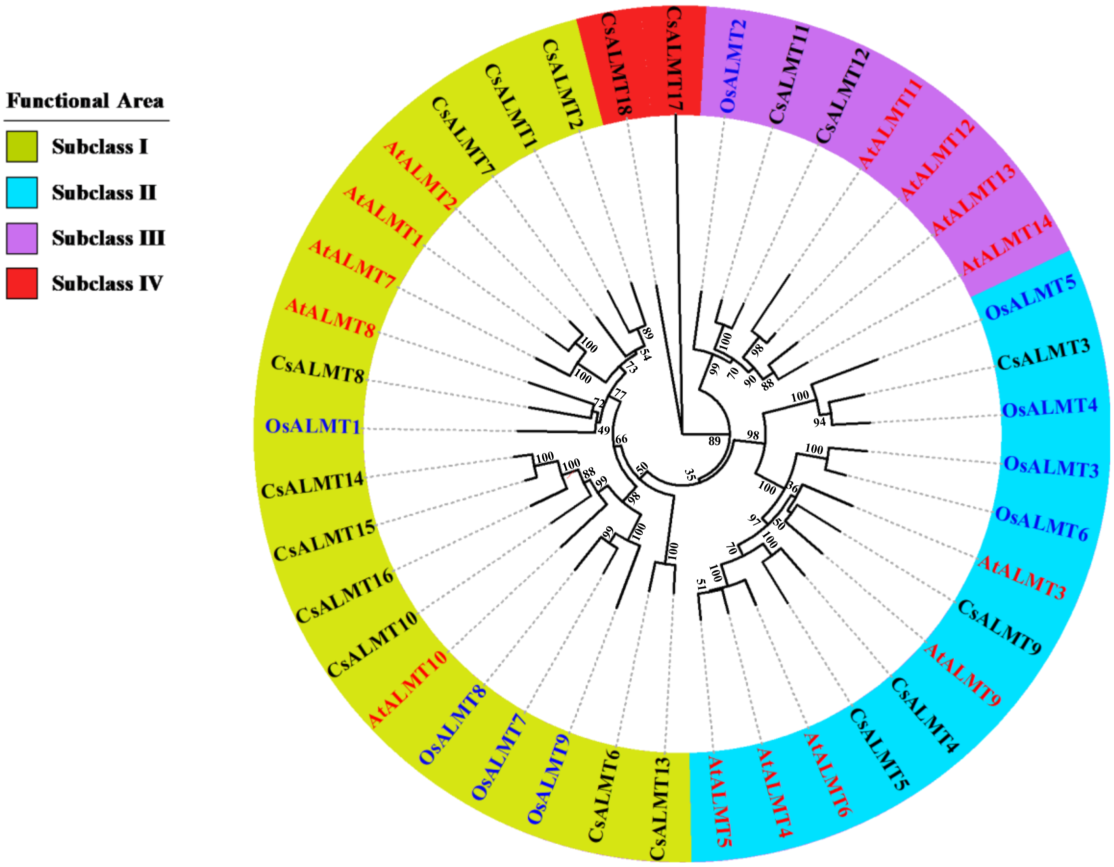

Figure S2 Phylogenetic tree of CsALMTs.

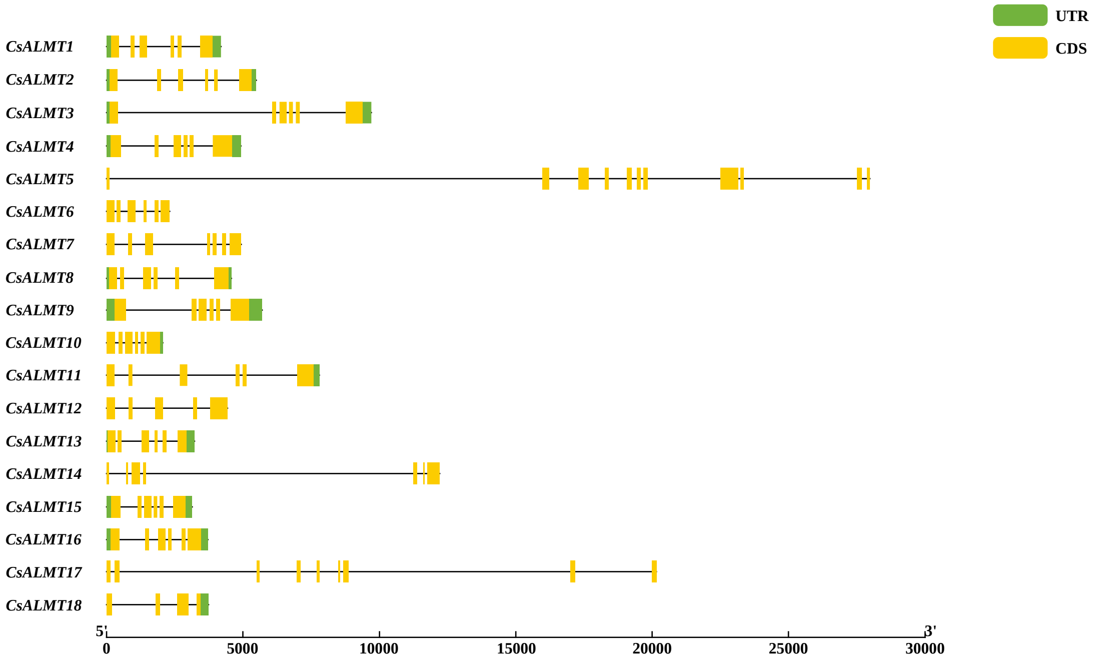

Figure S3 Gene structure of *CsALMTs*.

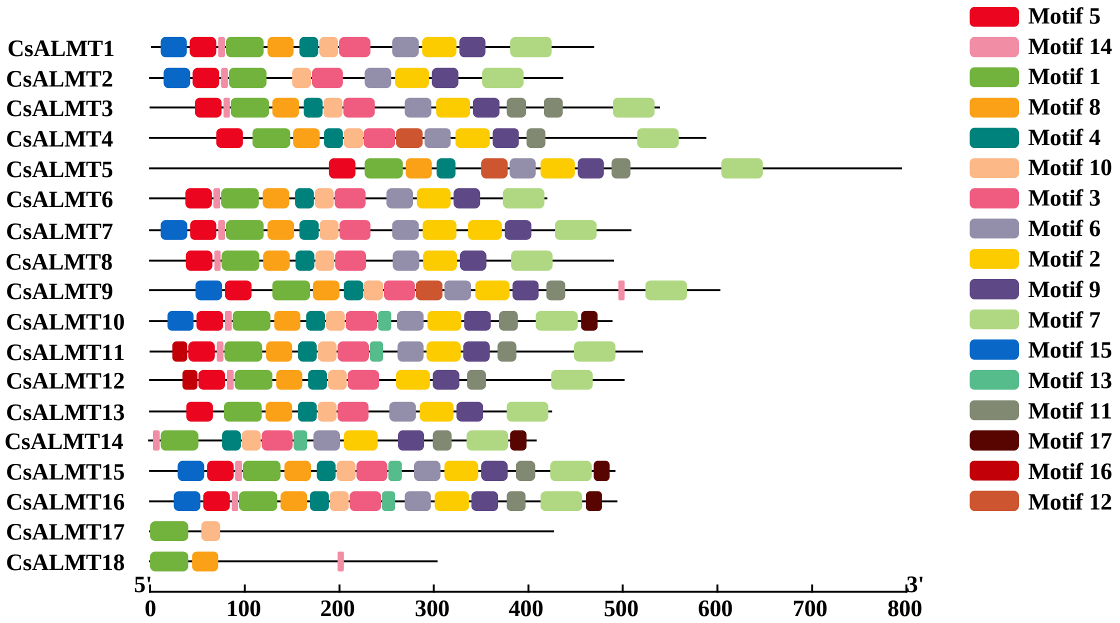

Figure S4 Motif analysis of CsALMTs.

Table S1 Bioinformatics analysis of *ALMT* genes in *Camellia sinensis*.

Gene name	Number of amino acids	Molecular weight	Theoretical pI	GRAVY	Instability index	Transmembrane number	Subcellular localization
CsALMT1	466	52037.32	8.13	0.07	40.40	5	plasma membrane
CsALMT2	435	47806.08	5.98	0.021	35.72	5	plasma membrane
CsALMT3	537	60066.99	6.26	0.003	31.18	6	endoplasmic
CsALMT4	586	65652.34	5.66	-0.009	40.16	5	endoplasmic
CsALMT5	793	88719.08	6.38	-0.036	41.93	5	endoplasmic
CsALMT6	418	46288.80	7.08	0.215	36.76	5	plasma membrane
CsALMT7	507	56762.84	8.61	0.059	28.24	5	plasma membrane
CsALMT8	489	53547.64	8.02	0.001	31.73	6	plasma membrane
CsALMT9	601	68675.16	5.96	-0.152	41.03	5	plasma membrane
CsALMT10	487	54423.09	5.72	0.034	31.00	6	plasma membrane
CsALMT11	519	58106.25	8.97	-0.041	38.39	5	plasma membrane
CsALMT12	500	55843.89	8.98	0.038	36.69	6	plasma membrane
CsALMT13	423	46017.59	6.96	0.202	34.24	5	plasma membrane
CsALMT14	408	45848.06	6.11	0.055	32.09	3	plasma membrane
CsALMT15	490	54672.54	6.53	0.067	32.10	6	plasma membrane
CsALMT16	492	54828.66	7.03	0.070	36.65	6	plasma membrane
CsALMT17	425	47099.03	5.93	0.359	37.33	5	extracellular
CsALMT18	302	32991.54	7.03	0.421	31.54	0	extracellular

Table S2 Second structure prediction of proteins encoded by *ALMT* genes in *Camellia sinensis*.

Pretein name	Alpha helix (%)	Beta turn (%)	Random coil (%)	Extended strand (%)
CsALMT1	61.80	3.43	24.25	10.52
CsALMT2	61.15	2.76	26.44	9.66
CsALMT3	57.73	2.05	29.61	10.61
CsALMT4	60.58	1.88	27.99	9.56
CsALMT5	51.83	5.30	29.63	13.24
CsALMT6	64.11	2.87	20.33	12.68
CsALMT7	59.37	4.34	24.06	12.23
CsALMT8	58.28	2.86	27.81	11.04
CsALMT9	56.74	2.83	30.45	9.98
CsALMT10	58.93	3.49	27.31	10.27
CsALMT11	58.96	2.70	25.63	12.72
CsALMT12	61.00	2.80	25.20	11.00
CsALMT13	61.47	3.07	23.88	11.58
CsALMT14	60.54	3.43	27.21	8.82
CsALMT15	58.16	3.27	28.37	10.2
CsALMT16	57.32	2.85	29.27	10.57
CsALMT17	36.47	11.06	23.29	29.18
CsALMT18	54.30	6.62	24.50	14.57

Table S3 Multilevel consensus motifs observed in *CsALMTs*.

Motifs	E-value	Width	Site	Multilevel consensus sequence
1	6.4e-359	41	18	MWAVMTVVVVFEFSVGATLGKGLNRGJGTLLAGGLGVGAHE
2	4.2e-237	37	16	PWKQYLKIGAALRHCAYCIEALHGCJNSEIQAPPELR
3	5.7e-179	34	15	CILTSVLICPVWAGEDLHNLVVRNFEKLASSLEG
4	7.9e-148	21	15	YDYGAMIFJLTFSLVSVSGYR
5	7.5e-181	29	15	GKDDPRRVIHSLKVGLALTLVSLLYYFDP
6	3.5e-178	29	15	GYKSVLNSKSTEESLANFARWEPPHGRFK
7	1.9e-167	45	16	EQTAVELLEILPLATFASLLIEIVARLEKJVEEVEELAELAEFKE
8	2.7e-090	29	16	QSGGIFEPIJJGISVFJJGAAATYTRFIP
9	3.2e-093	29	15	FSDPCIRLSSECSKALKELALSIKTMTKS
10	1.2e-062	21	15	VDEVLEMAHQRLSTILIGTAI
11	5.0e-038	21	10	EVHEAVZELQNDLKSLPYLLL
12	4.4e-028	29	3	CVNGYLQCVEYVRVPSKILTYQASDDPIY
13	5.1e-011	15	5	CVAEYFNDDENANEQ
14	1.1e-009	8	14	YDGVGGNA
15	1.8e-006	29	7	GLFSRAWRRLKGLPEKLWSKVWEFLEKAW
16	8.7e-003	17	2	EKMKRLPGLTWKTIWKV
17	1.4e-002	18	3	DDEKSQQNQSNTEKIDDQ

Table S4 Primer sequences information for *ALMT* genes.

Gene name	Forward primer (5'-3')	Reverse primer (5'-3')
CsALMT1	ACTGTTGTCGTCGTCTTCG	CAATGGTTCGGTTGTCTC
CsALMT2	GCCAGCGTTGTGATTGT	CCTCGGCTCCCATTTT
CsALMT3	CCAATACCGCATCAAAGC	CCACGAATGTAGCCTCCT
CsALMT4	TCGCAAATCTCACTTACTACCG	AAGCCTCGCAACAAACTCAA
CsALMT5	GAAATACAGGCTCCACCG	CTTCCCAAGTTGCCGAGT
CsALMT6	ACAGCAGCAACATACTTTCG	GTTCATCACTAGCCCAGAT
CsALMT7	AAAGGCATAAACAGAGGA	GCTAGGAGGAAGACAAATA
CsALMT8	TGGTAACGGTGACGGTGATG	TTTGGCTAGTGTTTGGTTCCTG
CsALMT9	TCTTTGGCGACATTTACTTC	GTTTGCCGTTGTTCCTCTAT
CsALMT10	TTTCTCCACCCAAATCTCCTG	GTCAACAATGGCTTCAACACG
CsALMT11	CAACACCGCCCTCAAATC	GCCACTACCTCCACAAGCA
CsALMT12	AGGAGAAATCCAGGGAGGTA	GCCAACCCTACTTTGAATGA
CsALMT13	TCCAAGTCGAGCGATGAGT	GGATTTGATGAAGGCTGTCTA
CsALMT14	TTTTGAATACATAGTGGGTGC	ATGATAGCTTGAGACCGACAC
CsALMT15	TGGCGGGAATGCTATG	GAGACCGACACCAAACTG
CsALMT16	AACTCGCTACTGTTACCACC	CTTGAACTTGAACTCGGCTA
CsALMT17	TGGTACTCTTGGCGTTGG	GGTGGATAGCCTCCTGTG
CsALMT18	ATGATGTAGTAGAAGGGCACC	ACCTCAATGACAGATAACGG
β-actin	GCCATCTTTGATTGGAATGG	GGTGCCACAACCTTGATCTT
